# Stroke lesion size – Still a useful biomarker for stroke severity and outcome in times of high-dimensional models

**DOI:** 10.1016/j.nicl.2023.103511

**Published:** 2023-09-18

**Authors:** Christoph Sperber, Laura Gallucci, Daniel Mirman, Marcel Arnold, Roza M. Umarova

**Affiliations:** a Department of Neurology, Inselspital, University Hospital Bern, University of Bern, Bern, Switzerland; b Department of Psychology, University of Edinburgh, Edinburgh, United Kingdom

**Keywords:** Lesion volume, Outcome prediction, Machine learning, NIHSS, mRS, Sample size

## Abstract

**Background:**

The volumetric size of a brain lesion is a frequently used stroke biomarker. It stands out among most imaging biomarkers for being a one-dimensional variable that is applicable in simple statistical models. In times of machine learning algorithms, the question arises of whether such a simple variable is still useful, or whether high-dimensional models on spatial lesion information are superior.

**Methods:**

We included 753 first-ever anterior circulation ischemic stroke patients (age 68.4±15.2 years; NIHSS at 24 h 4.4±5.1; modified Rankin Scale (mRS) at 3-months median[IQR] 1[0.75;3]) and traced lesions on diffusion-weighted MRI. In an out-of-sample model validation scheme, we predicted stroke severity as measured by NIHSS 24 h and functional stroke outcome as measured by mRS at 3 months either from spatial lesion features or lesion size.

**Results:**

For stroke severity, the best regression model based on lesion size performed significantly above chance (p < 0.0001) with R^2^ = 0.322, but models with spatial lesion features performed significantly better with R^2^ = 0.363 (t(7 5 2) = 2.889; p = 0.004). For stroke outcome, the best classification model based on lesion size again performed significantly above chance (p < 0.0001) with an accuracy of 62.8%, which was not different from the best model with spatial lesion features (62.6%, p = 0.80). With smaller training data sets of only 150 or 50 patients, the performance of high-dimensional models with spatial lesion features decreased up to the point of being equivalent or even inferior to models trained on lesion size. The combination of lesion size and spatial lesion features in one model did not improve predictions.

**Conclusions:**

Lesion size is a decent biomarker for stroke outcome and severity that is slightly inferior to spatial lesion features but is particularly suited in studies with small samples. When low-dimensional models are desired, lesion size provides a viable proxy biomarker for spatial lesion features, whereas high-precision prediction models in personalised prognostic medicine should operate with high-dimensional spatial imaging features in large samples.

## Highlights

• Stroke lesion size is a common imaging marker to predict or explain stroke outcomes.
• High-dimensional spatial features predicted stroke severity only slightly better.
• Lesion size and spatial features were comparable predictors for functional outcome.
• Lesion size is a viable biomarker in small samples or simple statistical models.
• For optimal prognostic precision, spatial imaging features should be utilised.

## Graphical abstract

**Figure:**
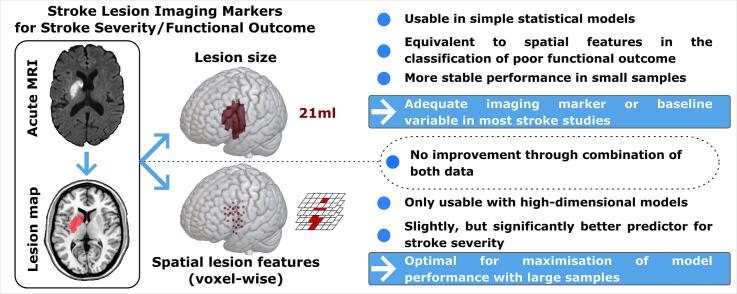

## 1 Introduction

In recent decades, new imaging techniques have expanded our knowledge of the functional anatomy of the brain and introduced a myriad of potential imaging biomarkers for stroke outcome. These include structural lesion imaging (Hope et al., 2013, Rondina et al., 2017), connectivity measures (Bonilha et al., 2014, Kuceyeski et al., 2016, Griffis et al., 2019, Siegel et al., 2016, Bowren et al., 2022), and measures of white matter integrity (Jokinen et al., 2005, Buch et al., 2016, Röhrig et al., 2022). The generation of these biomarkers often requires specialised imaging sequences or methodological pipelines, and the resulting data are often high-dimensional meaning that only complex machine learning algorithms can handle them.

The volumetric size of a brain lesion stands out among this group of biomarkers. The estimation of lesion size is possible with standard clinical protocol imaging and the required methodological steps are relatively few. Most importantly, lesion size is only a single variable that can be used in simple statistical models. Hence, it is not surprising that lesion size is an often used proxy of stroke severity in the evaluation of stroke outcome and post-stroke cognitive deficits (Goldenberg and Spatt, 1994, Pan et al., 2006, Minnerup et al., 2010, Long et al., 2011, Kaczmarczyk et al., 2012, Vogt et al., 2012, Laredo et al., 2018). Beyond, lesion size often serves as a baseline predictor, on top of or against which the predictive value of new biomarkers is evaluated (Bahrainwala et al., 2014, Benghanem et al., 2019, Carlson et al., 2020, Gialanella et al., 2013, Hope et al., 2013, Kuceyeski et al., 2016, Loughnan et al., 2019, Umarova et al., 2019, Zhu et al., 2010). However, in times of machine learning algorithms, model predictors may include high-dimensional spatial lesion information, such as voxel-wise topographies of the lesion area. Collapsing imaging information into a single variable, such as lesion size or a unidimensional representation of lesion location by pre-defined regions of interest, as in lesion load of the corticospinal tract (Zhu et al., 2010), is not required to compute statistical or predictive models. High-dimensional models might allow modelling algorithms to retain more complex information that cannot be represented by a single variable and that might be required to accurately predict stroke outcomes. The question remains if lesion size – a one-dimensional metric derived entirely from lesion imaging – is still a useful biomarker, or whether it is a methodological atavism from times when statistical models were incapable of processing high-dimensional imaging features. There are compelling conceptual and statistical reasons both for and against lesion size as a biomarker.

### 1.1 Why should lesion size be obsolete in times of high-dimensional models?

*Brain functions are localised*: Classical cortical localisationism has meanwhile been recast into a modern theory that assumes fibre connections and large-scale networks underlie brain functions (Catani et al., 2012). The assumption that brain functions are localised to certain brain structures or networks is a largely undisputed paradigm of cognitive neuroscience. If brain functions are localised, and not holistically organised, lesion location should be able to explain and predict the emergence and recovery of cognitive deficits.

*Redundancy of information*: Lesion size correlates highly with the lesion status in some brain regions (Sperber, 2022). Hence, the variance introduced by the variable lesion size already exists within the spatial lesion information on the level of single features and is partially redundant. Further, the information about lesion size fully exists within the spatial lesion information. A good multivariate modelling approach with sufficient training data should be able to derive potentially relevant information about lesion size from the lesion image without adding the variable explicitly.

### 1.2 Why should lesion size still be a relevant biomarker?

*The curse of dimensionality*: Clinical data are often noisy, have outliers, and samples might be small. Model training in a high-dimensional feature space may be doomed to create subpar results under such conditions. Lesion size contains less information than the entire spatial lesion data but may provide a proxy variable that is a robust biomarker even under suboptimal conditions.

*Explaining the oddballs*: Brain areas are damaged by lesions of typical size (Sperber and Karnath, 2016, Sperber, 2022), but there is still heterogeneity in the data. Imagine a voxel in the corticospinal tract that is typically damaged by large lesions. A lesion in this voxel might usually cause full disconnection and hemiparesis. However, a rather uncommon small lacunar lesion in this voxel might only partially disrupt the corticospinal tract and not cause a severe deficit. The interaction between the lesion size and spatial lesion information could allow models to account for this heterogeneity in atypical cases.

*Holism as a proxy:* Cognitive brain functions are often anatomically better understood on the level of large-scale networks (Siegel et al., 2016, Griffis et al., 2019), which should also be true for general stroke impairment or outcome (Cheng et al., 2023). If models with spatial lesion information struggle to represent the damage of such networks due to any reason – such as small sample sizes or the high-dimensional, multi-collinear structure of spatial imaging data – lesion size, as a holistic measure of brain damage, may serve as a possible rough proxy to explain critical impairment of functional large-scale networks.

*Pathophysiological effect of lesion size:* Stroke leads to a cascade of pathophysiological events that include a neuroinflammatory response that exacerbates blood–brain barrier damage, microvascular failure, brain oedema, and oxidative stress, and directly induces neuronal cell death further worsening stroke outcome (Shi et al., 2019, Stoll and Nieswandt, 2019). Acute stroke might cause autonomic dysfunction (Xiong et al., 2018) including stroke-heart syndrome (Scheitz et al., 2022). This cascade of inflammatory, autonomic, and humoral complications can be explained by local cerebral and systemic mediators which may be linked to lesion size.

### 1.3 Aims of the study

Given the conflicting arguments for and against the use of lesion size as a biomarker, we evaluated the predictive values of volumetric lesion size versus high-dimensional spatial lesion imaging features in stroke severity and outcome, as well as their potential complementary predictive value.

## 2 Methods

### 2.1 Study concept

In this study, we predicted stroke severity and functional stroke outcome in patients with a first-ever anterior circulation ischemic stroke from spatial lesion features (i.e. binary 3D maps that indicate the lesion area) or/and lesion size. We first optimised the out-of-sample prediction for either variable alone. For this aim, we evaluated numerous approaches, including non-linear algorithms and, for spatial lesion imaging data which included a large set of features, algorithms that are capable of processing high-dimensional data. Second, we combined the optimised models to evaluate the potential complementary value of both variables.

### 2.2 Patients and clinical assessment

**Fig. 1:**
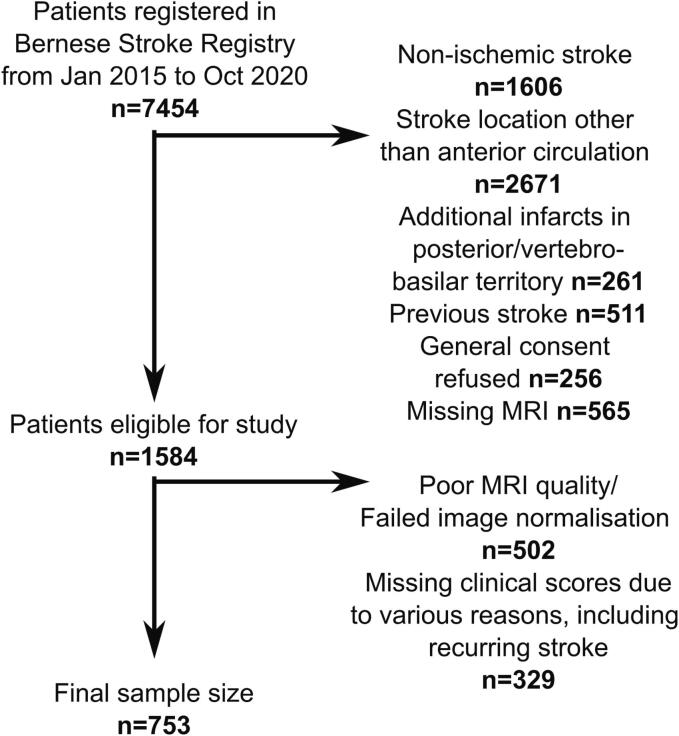
Recruitment flowchart.

This retrospective cohort study on clinical routine data investigated patients with ischemic stroke admitted to the Bern Stroke Centre between January 2015 and October 2020. The main inclusion criteria were a first-ever ischemic anterior circulation stroke and MRI acquired about 24 h after admission. A flowchart documenting exclusion criteria and recruitment stages is shown in Fig. 1. Written informed institutional general consent for research was available from all participants or their guardians. The study received approval from the local ethical committee (Kantonale Ethikkommission Bern KEK 2020–02273) and was conducted in accordance with the Declaration of Helsinki.

Stroke severity was assessed by the National Institutes of Health Stroke Scale (NIHSS) at 24 h after admission. We did not use NIHSS at admission, as patients underwent acute recanalization therapy and the final lesion load is rather reflected by NIHSS 24 h. Clinical and stroke data were recorded by the attending physician. Stroke outcome as measured by the modified Rankin Scale (mRS) at 3 months after stroke was either assessed within clinical follow-up examination or by telephone. In line with several previous studies (see Ganesh et al., 2018), we dichotomised the mRS into favourable (0–1) versus poor (≥2) outcome. This cutoff created more even groups and potentially allowed for better prediction performance compared to another common cutoff of 0–2 versus ≥ 3.

### 2.3 Brain imaging and lesion delineation

Lesion masks were delineated on diffusion-weighted MRI scans acquired about 24 h post-stroke as described previously (Umarova et al., 2014). At this time point, stroke-damaged brain tissue is usually demarcated and emerges as a hyperintense lesion in T2-weighted and diffusion-weighted MRI scans. Images were acquired at a slice thickness of 4–5 mm with a 1.5T or 3T Siemens scanner. We identified lesioned tissue by semiautomatic lesion segmentation using a region-of-interest toolbox in SPM12 (https://www.fil.ion.ucl.ac.uk/spm/software/spm12). Semiautomatic lesion segmentation has, compared to manual segmentation, a speed advantage, while user interaction still identifies possible errors and ensures high data quality (de Haan et al., 2015). Individual intensity thresholds were manually selected to best match the lesion map and the hyperintense diffusion-restricted brain tissue. Other available sequences (e.g., FLAIR, apparent diffusion coefficient maps) were consulted to ensure accurate lesion delineation. Lesion masks were then normalized to the standard Montreal Neurological Institute (MNI) space at 2x2x2 mm^3^ resolution with normalisation parameters from the coregistered T1 scans (Ashburner and Friston, 2005). We used the resulting binary lesion masks and their volumetric size as predictors in our current study.

### 2.4 Feature generation for lesion location

**Fig. 2:**
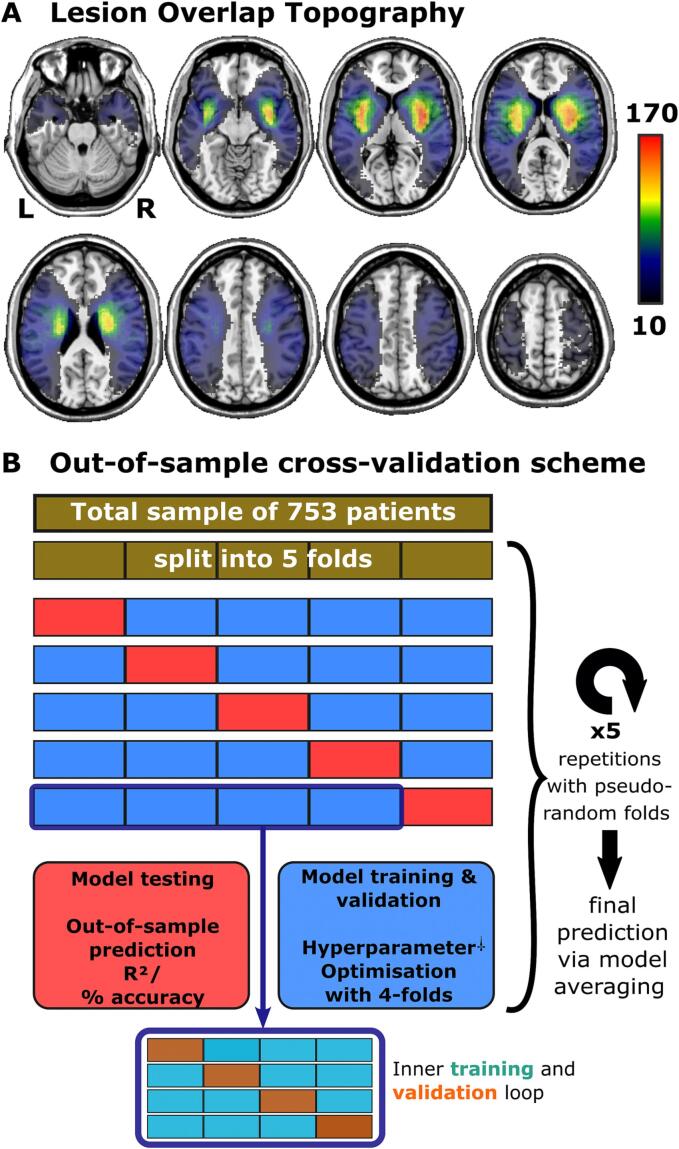
**Lesion topography and cross-validation scheme. (A)** Lesion overlap topography of all 753 lesions after normalisation. The voxels shown in the heatmap indicate the imaging space for spatial features used in model training. **(B)** Cross-validation scheme to assess model generalisability in out-of-sample predictions. † Some algorithms did not include hyperparameters and were simply trained on the 4 inner folds (blue). (For interpretation of the references to colour in this figure legend, the reader is referred to the web version of this article.)

The lesion masks with 79x95x79 voxels at 2x2x2 mm^3^ resolution included an excessively large set of features. We first limited the relevant data space to voxels lesioned in at least 10 patients (Fig. 2A), which removed voxels that provided little to no variance. For algorithms operating with spatial lesion features, the predictors were the binary status (“1″ lesioned/“0” intact) of each of the remaining 98,301 voxels. These were utilised either voxel-wise or after additional dimensional reduction by principal component analysis. The computational components derived from a principal component analysis can describe correlated data, such as a sample of voxel-wise lesion masks, with fewer variables. The components are related to the typical anatomy of stroke (Zhao et al., 2020) and can be used as efficient and effective predictors (Kasties et al., 2021). Conceptually, both voxel-wise and componential data still represent the same spatial lesion information. We further evaluated different sets of retained components, as a small set of components might exclude relevant features, and a large set might lead to over-fitting. We investigated components that cumulatively explained 40, 50, 60, 70, 80, and 90% variance in the lesion data. These sets contained between 5 and 246 components.

### 2.5 Analysis of prediction performance

We evaluated the performance of different prediction models with a nested cross-validation scheme (Fig. 2B), including 4 inner folds for training, hyperparameter optimisation and validation. Parameters were trained within these 4 folds and, for algorithms that included hyperparameters, these were optimised by 4-fold cross-validation. We tested the prediction performance of the trained and validated model in the remaining, held-out fifth fold. Predictions for the entire sample were obtained by repeating this procedure 5 times so that each of the five folds was the test fold once. The assignment to folds was pseudo-randomised with the same assignments across all models. We additionally repeated the entire nested cross-validation scheme five times with a randomised assignment to folds to control for performance fluctuations introduced by the assignment to folds. We averaged model performance for NIHSS severity and picked the majority decision for mRS classification across the five repetitions. The primary outcome variable was the coefficient of determination R^2^ for the NIHSS and the proportion of correct classifications for poor versus favourable stroke outcome at 3 months (mRS 0–1 versus ≥2).

In the first step, we evaluated several algorithms to optimise the prediction for either lesion size or spatial lesion features alone. The rationale for this optimisation was to ensure that any results were not driven by the inadequacy of an algorithm for certain data but by the predictive value of the data. We statistically compared the prediction performance between the nominally best models for spatial lesion features and lesion size by comparison of residuals with a paired *t*-test, respectively the proportion of correct classifications with a McNemar-Test. For the nominally best algorithms with low computational demands (e.g. logistic or polynomial regression), we tested if overall prediction performance was above chance by permutation. For 10.000 times, we repeated the prediction with permuted target variables to estimate the distribution of prediction performance scores under the null hypothesis. We implemented all analyses in Matlab R2022b with the Regression learner toolbox. Continuous predictor variables were standardised to z-scores.

#### 2.5.1 Regression of NIHSS 24 h

For regression of stroke severity based on spatial lesion features, we first tested *support vector regression* (SVR) and *Gaussian process regression* (GPR), two kernel-based methods. Both methods are prone to over-fitting with non-relevant lesion data (Rondina et al., 2017, Kasties et al., 2021). Therefore, we evaluated these algorithms across voxel-wise and componential data representations of lesion imaging data. We used Bayesian optimisation procedures to optimise hyperparameters for SVR with a linear, Gaussian or radial basis function kernel – with the hyperparameters box constraint C, kernel scale γ, and ε – and for GPR with a squared exponential or rational quadratic kernel – with the hyperparameters kernel scale, basis function and σ. Second, we tested *lasso regression* and *elastic net regression* with the predefined α-values of 1 and 0.5 and optimised the regularisation parameter λ across a geometric sequence of potential values. These closely related linear regression approaches apply to high-dimensional data as they utilise regularisation to identify a small set of relevant predictors. We evaluated these algorithms with voxel-wise data and the largest set of components. Third, we tested *partial least squares regression* (PLSR), a linear regression approach that inherently applies componential decomposition on both predictor and target data, on voxel-wise data with an optimised number of components. For lesion size, we evaluated simple linear regression, 2nd to 5th-degree polynomial regression, and SVR and GPR with non-linear kernels and the same parameters as for lesion location. Additional details on hyperparameters are reported in the supplementary.

#### 2.5.2 Classification of favourable versus poor stroke outcome at 3 months

For the classification of favourable versus poor stroke outcome based on spatial lesion features, we first tested *support vector machines* (SVM) with a linear, Gaussian or radial basis function kernel and box constraint C and kernel scale γ as hyperparameters. We applied SVM across voxel-wise and componential data representations of spatial lesion information. Second, we used bagged decision trees with the random forest algorithm and 100 trees per model on the componential features. For each split, sqrt(n) out of n features were randomly considered and the hyperparameter minimal leaf size was tuned by Bayesian optimisation. For lesion size, we evaluated only logistic regression.

#### 2.5.3 Evaluating the impact of sample size on model performance

In an additional experiment, we evaluated if sample size impacts the performance of lesion size version spatial lesion features as predictors. With the nominally best models based on lesion size or spatial lesion features in the previous analysis, we repeated the modelling procedures with a smaller sample. In the previous analysis, the 4 folds in the inner loop for training/validation included ∼600 patients. For the additional analysis, we repeated the analysis with ¼ and 1/12 of the original sample size, i.e. 150 or 50 patients, as a training/validation set and divided this sample into 4 new folds for training/validation. The out-of-sample test folds were left the same for comparability.

#### 2.5.4 Model combination

With the optimised models for spatial lesion features or lesion size alone at hand, we evaluated if both measures could be combined to obtain a superior model. We utilised two strategies: feature concatenation and model stacking. For feature concatenation, we added the variable lesion size as an additional predictor to the best model based on spatial lesion features. Model stacking, on the other hand, utilises the predictions of multiple models as predictors in a *meta*-learner, which can effectively combine neuroimaging biomarkers (Pustina et al., 2017). We used the two optimal prediction algorithms for spatial lesion features and size alone for prediction in the training/validation folds (i.e. within-sample) and used the two predictions as new predictors in a *meta*-learner model. Only then, the final prediction was evaluated in the test fold. As *meta*-learner, we again tested different applicable algorithms.

### 2.6 Data availability

Analysis scripts and prediction data are available at https://data.mendeley.com/datasets/k4gvdffhjg/2 for exact documentation of the algorithms. Clinical data are not publicly available, but qualified researchers may contact author R. Umarova (roza.umarova@insel.ch) to request access to anonymised data. Proposals need to be approved by the local ethics commission.

## 3 Results

**Fig. 3:**
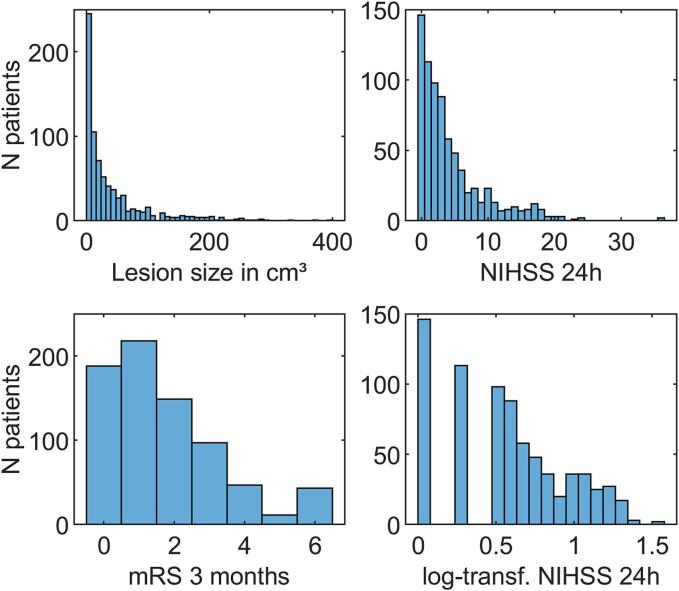
**Data distribution of main study variables.** Histogrammes show the distribution of the main study variables. The log_10_(x + 1) NIHSS scores (lower right panel) were used in the prediction algorithms.

The final sample included 753 patients with an average lesion size of 41.9 cm^3^ (±58.5; range 0.1–397.4). Fig. 3 shows the distribution of the main study variables. Table 1 shows demographic and clinical data. As the NIHSS scores were strongly skewed, we de-skewed this variable with a log_10_(x + 1) transformation. 347 out of 753 patients (46.1%) showed poor outcome as indicated by a 3-month mRS of ≥2.

**Table 1:** Demographic and clinical data.

All participants, N = 753	
Age, years mean (SD; range)	68.4 (15.2; 18–97)
Sex, Male %	54.5
History of transient ischemic attack, %	4.4†
Hypertension, %	68.4†
Diabetes, %	15.7†
Smoking, %	27.1†
Hyperlipidemia, %	67.1†
Atrial Fibrillation, %	27.5†
Coronary Heart Disease, %	19.2†
Body-Mass Index, mean(SD)	26.6 (4.8) †
NIHSS 24 h, mean(SD; range)	4.4 (5.1; 0–36)
mRS 3 months, median(quartiles)	1 (0.75; 3)
mRS favourable (0–1) versus poor (≥2), N	406; 347

Demographic or clinical variables marked with ‘†’ contained missing values (not more than 11.6% of the total sample) which were omitted in the computation of characteristics. SD – standard deviation; NIHSS – National Institutes of Health Stroke Scale; mRS – modified Rankin Scale.

### 3.1 Prediction results of stroke severity at 24 h

**Fig. 4:**
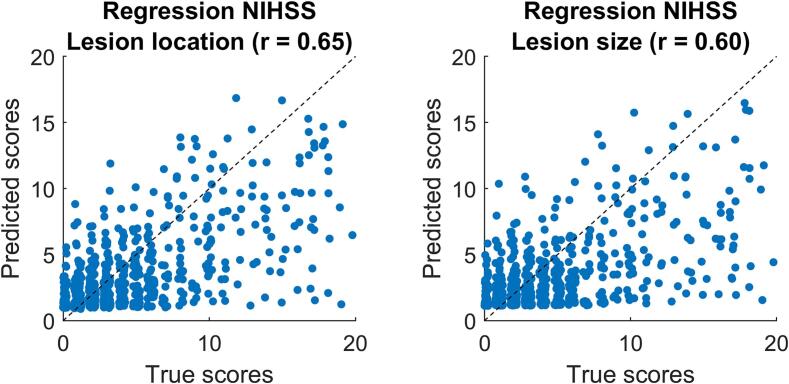
**Regression results on NIHSS.** Scatter plots of true and predicted NIHSS 24 h scores after re-transformation of log-transformed scores. Results are taken from the best algorithm for lesion location (support vector regression with voxel-wise spatial lesion features) and the best algorithm for lesion size (polynomial regression) as further described in the text. A few outliers with higher true scores were omitted. True scores were slightly jittered around the integer values in x-direction to allow for the visualisation of adjacent data points.

The best prediction of NIHSS 24 h based on spatial lesion features was achieved by an SVR with radial basis function kernel applied on voxel-wise lesion data with R^2^ = 0.363 and a correlation between actual and predicted scores of r = 0.61 for log-transformed scores respectively r = 0.65 for the original non-transformed scores (Fig. 4A). The best prediction based on lesion size was achieved by a 4th-degree polynomial regression with an R^2^ = 0.322 and a correlation between actual and predicted scores of r = 0.57, respectively r = 0.60 with the original scores (Fig. 4B). Table 2 reports results across model algorithms.

**Table 2:** Main results – spatial lesion features versus lesion size.

NIHSS – Spatial features	R^2^	NIHSS – Lesion size	R^2^
SVR	**0.363**	SVR	0.318
GPR	0.353	GPR	0.317
Lasso Regression	0.294	Linear regression	0.287
Elastic Net regression	0.298	Polynomial regr.	**0.322**
PLSR	0.314		

mRS – Spatial features	Acc.	mRS – Lesion size	Acc.
SVM	**62.6 %**	Logistic Regression	**62.8 %**
Random Forest	61.6 %		

Out-of-sample prediction accuracy for models based on either lesion size or spatial lesion features. The R^2^ values were computed based on the average out-of-sample prediction for each patient and accuracy values based on the majority decision, each across 5 repetitions of the modelling procedure. For algorithms that have been used with different parameters or data features, only the results of the best-performing model are shown. Note that chance classification performance for mRS was 53.9% due to the slightly uneven groups of 406 against 347 patients. Detailed results across models are reported in Supplementary Tables 1 and 2. R^2^ – coefficient of determination; Acc. – Classification accuracy.

The absolute out-of-sample prediction residuals for the best model based on spatial lesion features were lower than for the best model based on lesion size (t(752) = 2.889; p = 0.004), meaning that the spatial lesion features model performed significantly better. Further, due to the low computational requirements for polynomial regression, we were able to test this model against chance level by permutation inference, which suggested that the prediction was highly significant above chance (p < 0.0001).

### 3.2 Prediction of stroke outcome at 3 months

The best classification of favourable versus poor mRS based on spatial lesion features was achieved by an SVM on componential data with a classification accuracy of 62.6%. The classification based on lesion size by logistic regression achieved a classification accuracy of 62.8% which did not significantly differ from the spatial lesion features model (p = 0.80). Permutation inference showed that the classification accuracy of the logistic regression was highly significant above chance (p < 0.0001).

### 3.3 The impact of sample size

With a decreased sample size for training/validation to only ¼ of the size of the previous experiment (150 patients), spatial lesion features with the previous best model were able to predict NIHSS 24 h with R^2^ = 0.340. Lesion size with the previous best model achieved an R^2^ = 0.319 which was not longer significantly different from the prediction performance with spatial lesion features ((t(752) = 1.265; p = 0.21). With the even smaller sample of 1/12 of the original size (50 patients), models on spatial lesion features predicted with R^2^ = 0.276, while lesion size dropped to chance level with the 4th-degree polynomial regression. We repeated the prediction with the other linear or polynomial regression approaches and found that linear regression still achieved R^2^ = 0.280, which was not different from spatial lesion features (t(752) = 1.147; p = 0.25), and that 2nd-degree polynomial regression even achieved R^2^ = 0.307, which, however, was still not significantly superior to spatial lesion features (t(752) = 1.333; p = 0.18).

The classification of favourable versus poor functional outcome at 3 months with only ¼ of the original sample size did, again, not differ between spatial lesion features (accuracy = 61.1%) and lesion size (accuracy = 62.0%; p = 0.52) with the previously best models. With an even smaller sample of 1/12 the original size, however, lesion size (accuracy = 61.1%) was a significantly better predictor than spatial features (accuracy = 56.6%; p = 0.005).

### 3.4 Prediction results after the combination of spatial lesion features and lesion size

The prediction performance did not improve with the combination of both data modalities. For stroke severity, the concatenated model with an SVR on spatial lesion features and lesion size achieved an R^2^ = 0.353 (compared to R^2^ = 0.363 for the model with spatial lesion features alone). Likewise, the best *meta*-learner with linear regression was numerically inferior with R^2^ = 0.341. For stroke outcome, the concatenated model with an SVM on componential spatial lesion features and lesion size reached a prediction accuracy of 62.0% (compared to 62.8% for the logistic regression on lesion size). For model stacking, we used the best random forest model instead of the SVM as a first-layer model of spatial lesion information, as the output of an SVM is binary, whereas random forests generate continuous probabilistic scores. The best *meta*-learners were SVM and random forests, both with 58.0% prediction accuracy.

## 4 Discussion

Brain lesion size is a comparatively simple stroke imaging biomarker that is entirely derived from a lesion image. The availability of high-dimensional machine learning algorithms might lead one to believe that such a simple derivate of the more complex three-dimensional lesion image has become an obsolete variable. In general, our present findings support this position, as prediction models based on spatial lesion features were equivalent to or better than models based on lesion size. However, the predictive value of lesion size was equivalent in the classification of stroke outcome and significantly, but still only modestly inferior in the prediction of stroke severity. This was especially surprising as the best prediction models using lesion size were simple, low-dimensional regression models. Therefore, the use of lesion size as a baseline predictor for stroke outcome or severity appears to be justified, especially when one desires a simple statistical analysis design that requires low-dimensional data. A likely explanation for this finding is the curse of dimensionality: even though spatial lesion features contain much more information than lesion size, the typical structure of spatial lesion imaging data is too complex to be adequately represented with the algorithms and sample sizes typical in stroke studies.

In the current study, we predicted stroke severity and outcome, which represent general stroke impact. On the other hand, many biomarker studies aim to predict specific cognitive or behavioural post-stroke deficits, such as aphasia or paresis (e.g. Hope et al., 2013, Bonilha et al., 2014, Buch et al., 2016, Kuceyeski et al., 2016, Rondina et al., 2017). As these functions can be localized to specific brain structures or networks, the value of lesion size as a biomarker may well be lower in this context. A good example comes from the prediction of aphasia, where lesion size is a decent biomarker for generalised aphasic deficits, while specific sub-deficits such as speech recognition are better predicted by lesion location (Thye and Mirman, 2018). This argument may explain that the prediction of stroke severity, but not stroke outcome was significantly better with spatial lesion features in our study. The mRS score three months after stroke represents a general functional outcome measure, which is affected by many non-anatomical clinical and demographic factors. On the other hand, the acute NIHSS score can be traced back to a smaller set of cognitive or motor domains that, in parts, can be localised to specific lesion locations (Cheng et al., 2023). Besides, the impact of time since stroke might explain the difference in prediction performance for NIHSS 24 h and mRS 3 months post-stroke. Predicting non-acute deficits after stroke may be more difficult in general, because additional factors such as plasticity, recovery, and rehabilitation come into play. Therefore, the predictive value of imaging markers may decrease with time since stroke, and any potential of high-dimensional models to better process these imaging markers may be lost with a patient’s transition to the chronic stroke stage.

A major limitation of the potential translational application of lesion size as a biomarker remains in light of potential future methodological refinements and the collection of large data sets by research consortia. Being only a single variable, lesion size leaves little to no room for improvements in feature engineering or model training. For spatial lesion information, predictions might be improved by the selection of the most informative features (Rondina et al., 2017) or a feature representation that summarises features in meaningful entities, for example, according to secondary data including reference connectome data (Bonilha et al., 2014, Kuceyeski et al., 2016). Further, larger training samples might improve model generalisability, especially for neural networks that require very large samples but inherently apply feature selection and generation. Hence, potential future translational applications of biomarkers in rehabilitation guidance and the prediction of outcome and recovery will likely not rely on lesion size as a biomarker.

Training of high-dimensional models might require large samples, and any advantage of spatial lesion features might only be realized with large samples as in the current study. In line with this assumption, we found that the advantage of spatial lesion features in the regression of stroke severity decreased with lower sample sizes to the point of not being significantly superior to lesion size anymore. For the classification of stroke outcome, lesion size even outperformed spatial lesion features for the smallest sample size. Thus, lesion size appears to be a particularly good biomarker for small sample sizes (in the current study ∼50 samples for training) although the performance of high-dimensional models may not necessarily be inferior here. In conclusion, the use of lesion size as a variable in studies with small samples and with simple statistical models appears to be justified.

Is there a genuine impact of lesion size on stroke outcome independent of lesion location, for example through a link to pathophysiological processes? This is a question that our study cannot answer. The combination of lesion size and spatial lesion features did not improve predictions. A possible reason might be that lesion size does not have a role that carries an inherent informative value. But, likewise, lesion size might have an inherent informative value that is redundant, as the information may already be contained within the spatial lesion features on the level of single features or the entire model.

### 4.1 Limitations

In the current study, we compared the predictive value of lesion size with the predictive value of the data from which lesion size was fully derived, i.e., the binary lesion images. However, other imaging data depicting anatomical brain impairment, such as disconnection data, better predict some cognitive deficits (e.g. Siegel et al., 2016, Griffis et al., 2019). For the prediction of several specific clinical outcome measures or cognitive deficits, disconnectomic imaging markers were previously found to outperform lesion size (Kuceyeski et al., 2016, Talozzi et al., 2023). Therefore, the value of lesion size as a biomarker will be lower when high-dimensional biomarkers superior to lesion images are available. Further, we analysed a sample with anterior circulation stroke only. The predictive value of topographic lesion data might be significantly higher in more heterogeneous samples including posterior circulation strokes. However, the lesion coverage in our study is comparable or even superior to many other studies on stroke outcomes and lesion anatomy (e.g. Kuceyeski et al., 2016, Cheng et al., 2023, Ernst et al., 2018) and our results should be representative of a large majority of cerebral strokes (compare to Ng et al., 2007).

Acute stroke imaging is limited in identifying the extent of the final stroke core (Goyal et al., 2020) and even in the chronic stage, structural changes of the lesion visible in MRI take place (Seghier et al., 2014). Hence, the inclusion of predictors derived from additional imaging at a later time point might further improve the prediction of clinical outcomes at 3 months. However, this would only have value for understanding brain pathology, but obviously not for prognostic applications.

### 4.2 Conclusions and perspective

High-dimensional machine learning algorithms using either spatial lesion features or lesion size as biomarkers were equivalent in the prediction of stroke outcome, and spatial features were slightly better than lesion size in the prediction of stroke severity. For the translational application in precision medicine that operates with large samples, spatial lesion information appears to be the optimal biomarker. Especially if future large consortia databases should allow the training of clinical prediction algorithms on many thousands of observations, high-dimensional models should be prioritised. Still, predictions based on lesion size were not markedly inferior and can be justified as a proxy for spatial lesion features in simple, low-dimensional statistical models. The combination of lesion size and spatial lesion features in a single model does not benefit prediction performance. Hence, lesion size appears to provide information that is non-complementary or redundant to spatial lesion information. The question remains how well our findings on general clinical post-stroke measures transfer to specific post-stroke cognitive deficits.

## CRediT authorship contribution statement

**Christoph Sperber:** Conceptualization, Methodology, Formal analysis, Writing – original draft. **Laura Gallucci:** Investigation, Data curation, Writing – review & editing. **Daniel Mirman:** Conceptualization, Writing – review & editing. **Marcel Arnold:** Resources, Investigation, Writing – review & editing. **Roza M. Umarova:** Conceptualization, Investigation, Data curation, Resources, Supervision, Writing – review & editing.

## Declaration of Competing Interest

The authors declare that they have no known competing financial interests or personal relationships that could have appeared to influence the work reported in this paper.

## Data Availability

Analysis scripts and prediction data are available at https://data.mendeley.com/datasets/k4gvdffhjg/2
