# Supplementary material for: Stroke lesion size – Still a useful biomarker for stroke severity and outcome in times of high-dimensional models
Source: Neuroimage Clin. 2023 Sep 18;40:103511. doi: 10.1016/j.nicl.2023.103511 (PMC10520672; doi:10.1016/j.nicl.2023.103511)
Supplement: Supplementary data 1 [file mmc1.pdf]

Stroke lesion size – still a useful biomarker for stroke severity and outcome in times of high-dimensional models

Supplementary

Supplementary Table 1a – Detailed results in the regression of stroke severity across models

Detailed regression results for stroke severity across conditions. The R^2 values were computed based on the average out-of-sample prediction for each patient across 5 repetitions. For algorithms using spatial lesion data, the 'Data' column indicates the data format, which was either full voxel-wise or processed by principal component analysis (PCA). The label indicates the per cent of explained variance by the PCA-derived features, e.g. 'PCA40' included the PCA components that cumulatively explained 40% of the total variance. 'Spatial' models were based on spatial lesion information, contrary to models based on lesion size. SVR – support vector regression; rbf - rbf – radial basis function kernel; GPR – Gaussian process regression; rat. qu. – rational quadratic kernel; squ. exp. – squared exponential kernel

Data	NIHSS - Spatial	R^2	NIHSS - Spatial	R^2
PCA40	SVR linear	0.284	SVR rbf	0.304
PCA50	SVR linear	0.319	SVR rbf	0.326
PCA60	SVR linear	0.319	SVR rbf	0.347
PCA70	SVR linear	0.327	SVR rbf	0.360
PCA80	SVR linear	0.198	SVR rbf	0.323
PCA90	SVR linear	0.068	SVR rbf	0.263
Voxel-wise	SVR linear	0.343	SVR rbf	0.363

Data	NIHSS - Spatial	R^2	NIHSS - Spatial	R^2
PCA40	SVR Gaussian	0.303	GPR rat. qu.	0.298
PCA50	SVR Gaussian	0.326	GPR rat. qu.	0.327
PCA60	SVR Gaussian	0.338	GPR rat. qu.	0.353
PCA70	SVR Gaussian	0.353	GPR rat. qu.	0.338
PCA80	SVR Gaussian	0.328	GPR rat. qu.	0.323
PCA90	SVR Gaussian	0.243	GPR rat. qu.	0.280
Voxel-wise	SVR Gaussian	0.359	GPR rat. qu.	0.344

Data	NIHSS - Spatial	R^2
PCA40	GPR squ. exp.	0.297
PCA50	GPR squ. exp.	0.321
PCA60	GPR squ. exp.	0.329
PCA70	GPR squ. exp.	0.332
PCA80	GPR squ. exp.	0.135
PCA90	GPR squ. exp.	0.042
Voxel-wise	GPR squ. exp.	0

Supplementary Table 1b – Detailed results in the regression of stroke severity across models (continuation)

Data	NIHSS - Spatial	R^2	NIHSS - Spatial	R^2
PCA90	Elastic Net Regr.	0.280	Lasso Regr.	0.280
Voxel-wise	Elastic Net Regr.	0.298	Lasso Regr.	0.294

	NIHSS – Lesion Size	R^2	NIHSS – Lesion Size	R^2
	Linear Regr	0.287	SVR Gaussian	0.317
	Polyn. Regr. 2nd degree	0.316	SVR rbf	0.318
	Polyn. Regr. 3rd degree	0.320	GPR rat. quad.	0.317
	Polyn. Regr. 4th degree	0.322	GPR squ. exp.	0.313
	Polyn. Regr. 5th degree	0.318		

Supplementary Table 2 – Detailed results in the classification of stroke outcome across models

Detailed classification results for stroke outcome with machine learning models across conditions. The accuracy values were computed based on the majority decision for each patient across 5 repetitions. For details see the legend of supplementary table 1. All results shown here originate from models on spatial lesion information. SVM – support vector machine; rbf – radial basis function kernel

Data	mRS - Spatial	Acc.	mRS - Spatial	Acc.
PCA40	SVM linear	60.7 %	SVM rbf	61.4 %
PCA50	SVM linear	62.4 %	SVM rbf	61.9 %
PCA60	SVM linear	62.6 %	SVM rbf	62.2 %
PCA70	SVM linear	60.4 %	SVM rbf	61.9 %
PCA80	SVM linear	58.7 %	SVM rbf	61.0 %
PCA90	SVM linear	56.2 %	SVM rbf	62.6 %
Voxel-wise	SVM linear	61.5 %	SVM rbf	62.0 %

Data	mRS - Spatial	Acc.	mRS - Spatial	Acc.
PCA40	SVM Gaussian	60.7 %	Random Forest	61.1 %
PCA50	SVM Gaussian	61.6 %	Random Forest	59.6 %
PCA60	SVM Gaussian	62.3 %	Random Forest	60.0 %
PCA70	SVM Gaussian	61.6 %	Random Forest	61.6 %
PCA80	SVM Gaussian	62.3 %	Random Forest	59.9 %
PCA90	SVM Gaussian	61.6 %	Random Forest	60.2 %
Voxel-wise	SVM Gaussian	61.8 %		

Additional details on hyperparameters in prediction algorithms

Support vector machines and Gaussian process regression were optimised with a Bayesian optimisation procedure with hyperparameters taken from default ranges of the MATLAB functions.

Support vector machine classification/regression: The box constraint (also known as soft margin C) was chosen among positive values log-scaled in the range [1e-3,1e3]. Kernel Scale (also known as γ) in non-linear kernels was chosen among positive values log-scaled in the range [1e-3,1e3]. ε in support vector regressions was chosen among positive values log-scaled in the range [1e-3,1e2]*IQR(Y)/1.349.

Gaussian Process Regression: basis function was selected as either 'constant', 'none', 'linear', or 'pure quadratic'. Kernel scale was chosen among real values in the range [1e-3*MaxPredictorRange, MaxPredictorRange], where MaxPredictorRange = max(max(X) - min(X)).σ was chosen among real values in the range [1e-4, max(1e-3,10*ResponseStd)], where ResponseStd = std(y).

Random forests were optimised with a Bayesian optimisation procedure with the hyperparameter minimal leaf size to be split. This limits the minimum number of observations for a node to be still split. This parameter indirectly also limits tree depth, as small nodes (i.e. nodes with only a few observations) are not further split to generate nodes at the next depth level. The parameter was selected between 1 and 30.

For lasso and elastic net regression, the hyperparameter λ was optimised. Following the documentation of the lasso.m function, a geometric sequence of potential values was evaluated, with only the largest value able to produce B=0. Among this set of values, the one that created the sparsest model within one standard error of the minimum MSE was selected.

Supplementary Figures – Prediction performance across cross-validation folds

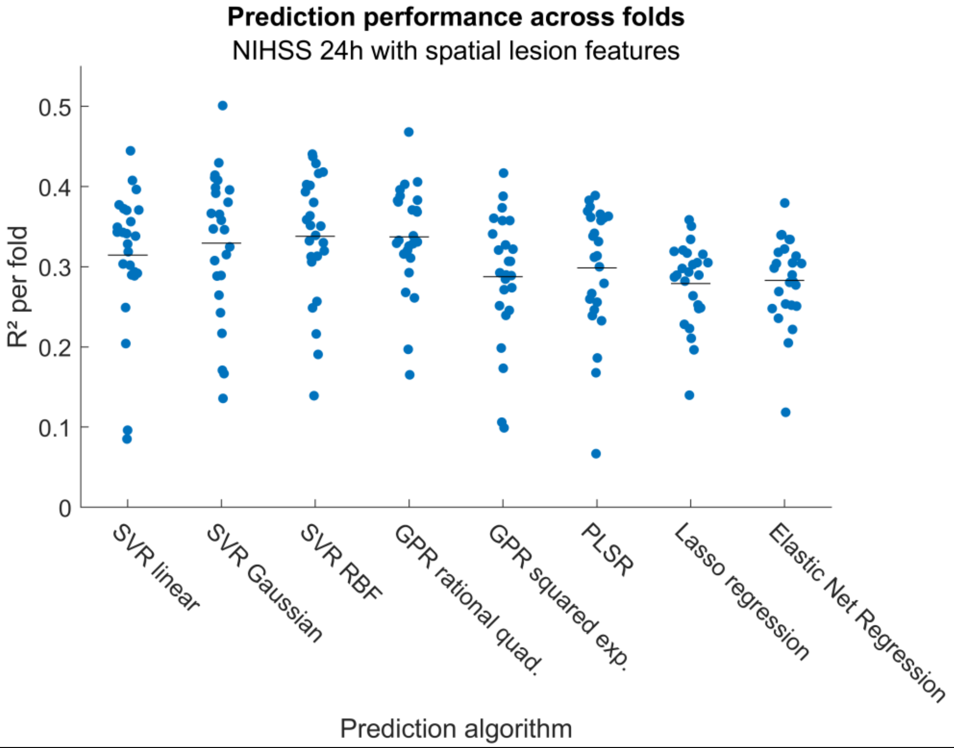

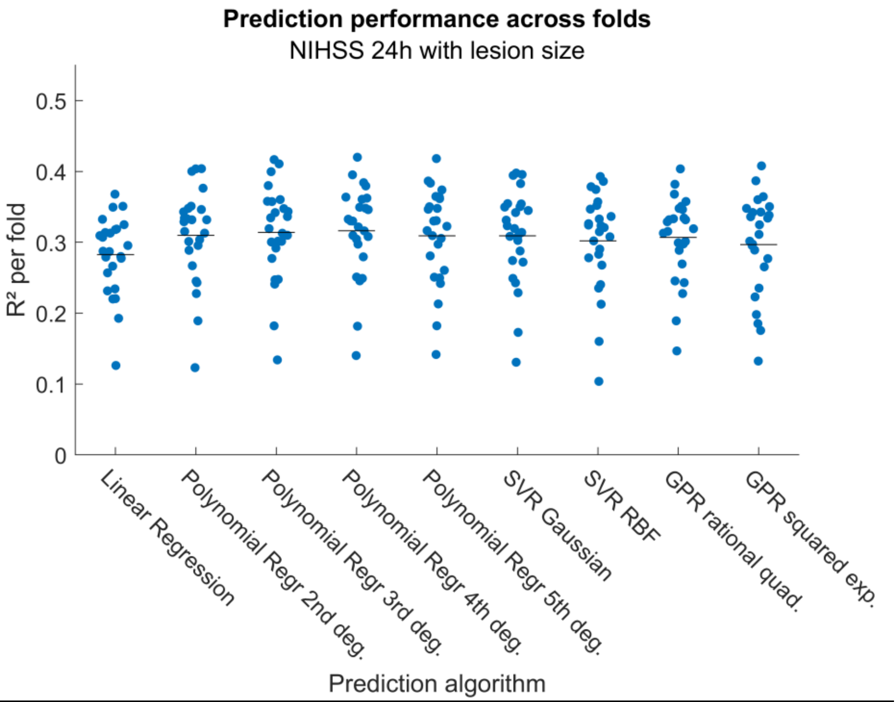

Supplementary Figure 1: Prediction performance in the regression of NIHSS 24h across all 25 folds (5 outer loop folds x 5 repetitions). Each dot represents one fold; the black bar indicates the mean performance. For algorithms with multiple sub-models (such as SVR with either voxel-wise or different componential data), only the best model is shown.

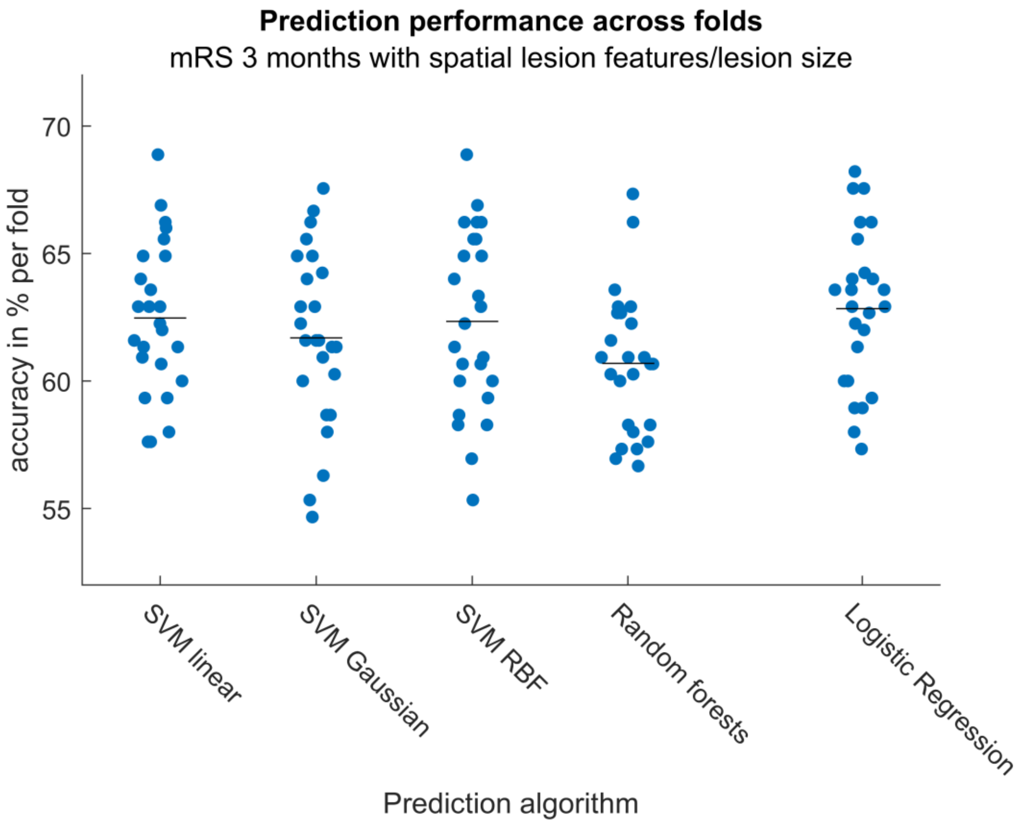

Supplementary Figure 2: Prediction performance in the classification of mRS 3months across all 25 folds (5 outer loop folds x 5 repetitions). Each dot represents one fold; the black bar indicates the mean performance. For algorithms with multiple sub-models (such as SVM with either voxel-wise or different componential data), only the best model is shown. The 4 models on the left used spatial lesion features, the 1 model on the right used lesion size.
